# Effect of Different Syrup Types on Turkish Delights (*Lokum*): A TD-NMR Relaxometry Study

**DOI:** 10.1021/acsfoodscitech.2c00222

**Published:** 2022-11-30

**Authors:** Pelin Pocan, Leonid Grunin, Mecit Halil Oztop

**Affiliations:** † Department of Food Engineering, Faculty of Engineering and Architecture, Konya Food and Agriculture University, 42080 Konya, Turkey; ‡ Department of Food Engineering, Middle East Technical University, 06800 Ankara, Turkey; § Resonance Systems GmbH, D-73230 Kirchheim unter Teck, Germany

**Keywords:** time-domain (TD) NMR relaxometry, magic sandwich echo (MSE), soft candies, food gels, allulose syrup

## Abstract

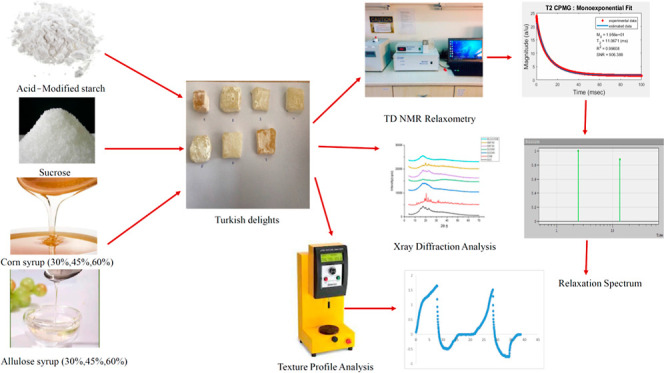

Turkish delights were formulated by using sucrose (control) and different types of corn syrups (having varying glucose/fructose ratios) and allulose syrup. 30% allulose syrup and 30% sucrose-containing Turkish delights were found to exhibit an amorphous structure. Time-domain NMR relaxometry experiments were also conducted on delights by measuring *T*_2_ relaxation times, and two distinct proton populations were observed in all formulations. The use of different syrup types at different substitution levels led to significant changes in the relaxation times (*T*_2a_ and *T*_2b_) of the samples, indicating that the relaxation spectrum might be used as a fingerprint for Turkish delights containing different types and amounts of syrup types. Second moment (*M*_2_) values which were measured from the signal acquired using a magic sandwich echo pulse sequence were also found to be an effective and promising indicator to detect the crystallinity of Turkish delights.

## 1 Introduction

Turkish delight (lokum) is a type of sugar-based jelly confectionery which contains starch as the gelling agent.^1^ As its name implies, it is a traditional confectionery product of Turkey and due to its economic value and market share in Turkey, it is protected under Turkish legislation covering ingredients and production methods. According to the detailed definition of Turkish legislation, Turkish delight (lokum) is a product which is prepared by mixing sugar, starch, drinking water, and citric acid or tartaric acid in appropriate amounts.^2^ For some types of Turkish delights, several types of seasonings or dried fruits can also be added to the lokum mixture as ingredients.^2^ Confectionery manufacturers might prefer to use corn syrup or other types of sweeteners instead of sucrose for various purposes such as to inhibit crystallization,^3^ to maintain the moisture of the products,^4^ and to decrease the cost.^5^ Corn syrup is generally used in the production of most confectionery products to prevent crystallization, improve their shelf life, and preserve the textural properties of the products during storage.^6^ However, the use of corn syrups [especially high-fructose corn syrups (HFCSs)] has been considered as a controversial issue. Some studies have hypothesized that the consumption of HFCS being more lipogenic than sucrose might increase the risk for dyslipidemia and nonalcoholic fatty liver disease.^7^

Therefore, as an alternative to these corn syrups, novel sweeteners have started to be used in food formulations. d-Allulose (formerly known as d-psicose) which is also classified as a rare sugar having 70% of the sweetness of sucrose and a caloric value of 0.39 kcal/g can be given as an example of these novel sweeteners with its promising health effects such as lowering blood glucose levels and reducing fat accumulation in the body due to its low calorie.^8^ In previous studies, d-allulose was used in the production of confectionery products such as gelatin-,^8,9^ starch-,^10^ and pectin^11,12^-based soft candies. In these studies, crystallization inhibition properties of d-allulose were found to be promising for the gelatin and starch-based soft candies.^8,10^ The use of allulose syrup in the production of Turkish delights might be also a choice of manufacturers due to its low caloric value and health benefits, as well as crystallization inhibition properties.

Time-domain NMR (TD-NMR) can be utilized to understand the type and amount of sugar (sucrose, allulose syrup, or corn syrup) in the formulations of Turkish delights as a promising tool due to its less laborious and noninvasive nature. In previous studies, relaxation times obtained through TD-NMR relaxometry were also used to explain the structural changes and water interaction within the food matrices. The *T*_1_ relaxation time is mostly associated with the crystal structures,^13^ whereas *T*_2_ relaxation times were used to detect changes in polymer–water and polymer–polymer interaction in gel systems^14−18^ in soft candy products.^8,10−12,19^ In these studies, multiexponential analysis of *T*_2_ relaxation data was found to be more useful to get an idea about the different proton pools found in the gel systems due to the multicompartment nature of food gels. Apart from gel systems and soft candy products, TD-NMR relaxometry was also utilized in various studies to characterize the dairy products such as milk,^20^ milk powder,^21^ ice cream,^22^ and yogurt.^23^ It has also been exploited widely to characterize the emulsions,^24^ meat products,^25^ and baked products such as gluten-free bread.^26^

In addition to these classical approaches which utilize *T*_1_ and *T*_2_ relaxation times [measured with the help of conventional TD-NMR sequences such as saturation recovery and Carr–Purcell–Meiboom–Gill (CPMG)], nonconventional methods such as magic sandwich echo (MSE) also seem to be promising to determine the changes in the microstructure of food samples. MSE might be defined as a refocusing sequence which could be applied prior to free induction decay (FID) detection, and it has been widely used in polymer science and the polymer industry.^27^ This technique was found to be useful to monitor polymer crystallization kinetics,^28,29^ to detect the crystallinity fraction and component mobility in polymers,^30^ and to investigate the changes in the structure of cellulose in terms of its crystal/amorphous fractions during water uptake.^31^ Recently, MSE has also started to be used in food applications such as investigating the crystallinity of different powder sugars,^32^ monitoring the structural changes occurring during the in vitro digestion of whey protein isolate hydrogels,^18^ and monitoring honey crystallization and melting processes.^33^ However, to the best of our knowledge, there is no study in the literature investigating the application of the MSE sequence on Turkish delights (*lokum*) to detect the structural changes related to the crystallinity of the samples. In the present work, *T*_2_ relaxation times obtained through conventional methods, as well as second moment (*M*_2_) values obtained by analyzing the MSE signal, which demonstrate the strength of hydrogen dipolar interactions within different samples,^34^ were utilized as a fingerprint to explain the changes among the samples that differ in terms of both sugar type and the amount of sugar/syrup.

The main objective of this study is to reveal the potential of the TD-NMR technique by using both conventional and nonconventional methods to detect the changes in Turkish delight samples containing different types and amounts of sugars/syrups, utilize TD-NMR to explain gel properties and solid–water interactions in Turkish delights, and examine the potential of the TD-NMR technique as an alternative to the widely used methods such as X-ray diffraction (XRD) by considering the crystallinity changes of different types of samples.

## 2 Materials and Methods

### 2.1 Materials

Sucrose (Bal Küpü, Aksaray, Turkey) was purchased from a local market in Ankara, Turkey. Corn syrups with commercial names (SCG40, SCG60, SRF30, and SMF42) were kindly provided by Sunar Mısır A.Ş (Adana, Turkey). Allulose syrup with the brand name “Wholesome” containing 5% glucose and 95% allulose was purchased from a local market in the USA. The total soluble solid content (TSSC, Brix) and glucose, allulose, or glucose/fructose content of these corn syrups are given in Table 1. Acid-modified starch was kindly provided by Kervan Gıda A.Ş (İstanbul, Turkey). Citric acid monohydrate was purchased from Sigma-Aldrich Chemical Co. (St. Louis, MO, USA). Distilled water was used in all formulations. The “COM” sample denotes a commercial Turkish delight that was purchased from a local market in Ankara, Turkey. Other types of Turkish delights were prepared in the laboratory.

**Table 1:** Specifications of Corn Syrups and Allulose Syrup Types That Were Used in the Production of Turkish Delights

syrup name	Brix (°)	glucose (%)	fructose (%)	allulose (%)
SCG40 (glucose syrup)	83	40		
SCG60 (glucose syrup)	82	60		
SRF30 (glucose/fructose syrup)	80	23	32	
SMF42 (glucose/fructose syrup)	70	51	42	
(allulose syrup)	77	5		70

### 2.2 Methods

#### 2.2.1 Preparation of the Samples

Turkish delights were prepared according to the method of Ilhan et al. (2020) with some modifications.^10^ COM and only sucrose-containing samples (SUC) were considered as control.

For the formulation of Turkish delight, 11 g of starch was mixed with 2 times the amount of water (22 g) by its weight and gelatinized in an oil bath at 140 °C for 5 min until it was dissolved completely. During this time, the sugar mixture and water were boiled up to 115 °C before being mixed with starch and water. 0.1 g of citric acid was also added to this sugar mixture for all formulations. Cooking was continued at 125 °C in an oil bath. Afterward, the mixture was poured into starch molds with dimensions of 2.5 × 2.5 × 2 cm and kept at room temperature (25 °C) for 48 h. Control Turkish delight samples (SUC) were prepared by using only powder sugar (sucrose), while other samples were prepared by using different types of corn syrups or allulose syrup with different substitution levels (30, 45, 60%) as the sugar source. They were classified with the same name together with the syrups that they contain (SCG40, SCG60, SRF30, SMF42, and allulose). The compositions (w/w) (%) of the Turkish delights are given in Table 2.

**Table 2:** Turkish Delights Formulated with Different Types of Sugar (Corn Syrup, Allulose Syrup, and/or Sucrose) (w/w) (%)

sample name	starch (%)	sucrose (%)	syrup (%)	citric acid (%)
COMTROL-1 (COM)	commercial product			
CONTROL-2 (SUC)	10	60		0.1
SCG40-30	10	30	30	0.1
SCG60-30	10	30	30	0.1
SRF30-30	10	30	30	0.1
SMF42-30	10	30	30	0.1
ALLULOSE-30	10	30	30	0.1
SCG40-45	10	15	45	0.1
SCG60-45	10	15	45	0.1
SRF30-45	10	15	45	0.1
SMF42-45	10	15	45	0.1
ALLULOSE-45	10	15	45	0.1
SCG40-60	10		60	0.1
SCG60-60	10		60	0.1
SRF30-60	10		60	0.1
SMF42-60	10		60	0.1
ALLULOSE-60	10		60	0.1

#### 2.2.2 TSSC Measurements

TSSCs of the slurries of the samples before cooking were measured by using a refractometer (HANNA HI 96801, HANNA Instruments, USA), and the results were reported as Brix (°) values.

#### 2.2.3 Moisture Content Determination

Moisture contents (MCs) of the different formulations were measured at 70 °C for 4 h in a vacuum oven (DAIHAN, Germany). Weight loss from the samples was recorded, and the MC of each sample was calculated on a wet basis.

#### 2.2.4 Color Analysis

L* (brightness), a* (red/green ratio), and b* (yellow/blue ratio) values of the Turkish delights were measured using a bench-top spectrophotometer [Datacolor 110 (Lawrenceville, NJ, USA)].

#### 2.2.5 Texture Profile Analysis

The texture profile analysis (TPA) test was performed by using a texture analyzer (Brookfield Ametek CT3, TA11/1000 probe, Middleboro, MA, USA) by following the method of Delgado and Bañón (2015) with some modifications. The samples were compressed twice with a cylindrical probe (25.4 mm in diameter). The testing conditions were *two consecutive cycles of 50% deformation, cross-head moved at a constant speed of 1 mm/s, and a trigger point of 0.05 N*.^35^ Hardness, adhesiveness, cohesiveness, springiness, and gumminess values of the Turkish delights were calculated by using TPA curves. The representative TPA curve is provided as the Supporting Information.

#### 2.2.6 TD-NMR Relaxometry Experiments

TD-NMR relaxometry measurements were conducted by using a 0.5 T (20.34 MHz) NMR instrument (Spin Track Resonance Systems GmbH, Kirchheim/Teck, Germany). *T*_2_ (spin–spin) relaxation times were measured for different formulations. For *T*_2_ measurements, the CPMG sequence was used with parameters of 100 μs echo time, 64 echoes, and 8 scans.

*T*_2_ data were analyzed by approaches as indicated in the study of Pocan et al. (2019). Non-negative least square analysis was conducted on *T*_2_ curves to obtain a relaxation spectrum. Relative areas (RAs; %), number, and amplitudes of peaks of the samples were recorded by using this method with XPFit (Softonics Inc., Israel).

As a nonconventional method, in order to detect the crystallinity of the samples MSE was used. The method from the study of Grunin et al. (2019) was followed.^32^ Crystallinity-related values were obtained by using second moments (*M*_2_), as explained by Grunin et al. (2019). For the measurement of second moment values, the MSE sequence was used with parameters of 10.000 ms repetition time and 16 scans. Before the crystallinity analysis by MSE, Turkish delight samples were exposed to drying at room temperature (25 °C) for 6 months to eliminate the excess moisture coming from the liquid portion of the sample, making it possible to get the signal coming from the only crystalline region as in the case study of Ozel et al. (2020).^18^

The final liquid fraction values (%) of the samples were also found by using the MSE sequence for the dried samples to get an idea about the final water fraction of the dried samples.

#### 2.2.7 Statistical Analysis

All measurements were carried out in replicates (two and three depending on the measurement) and reported as means and standard errors. Statistical analysis for syrup-containing samples was performed by analysis of variance (ANOVA) (Minitab Inc., Coventry, UK). For the comparison of results, Tukey’s comparison test was applied at a 95% confidence interval. The correlation coefficients were also expressed by Pearson correlation at a 95% confidence level.

## 3 Results and Discussion

### 3.1 TSSC Measurements

**Figure 1:**
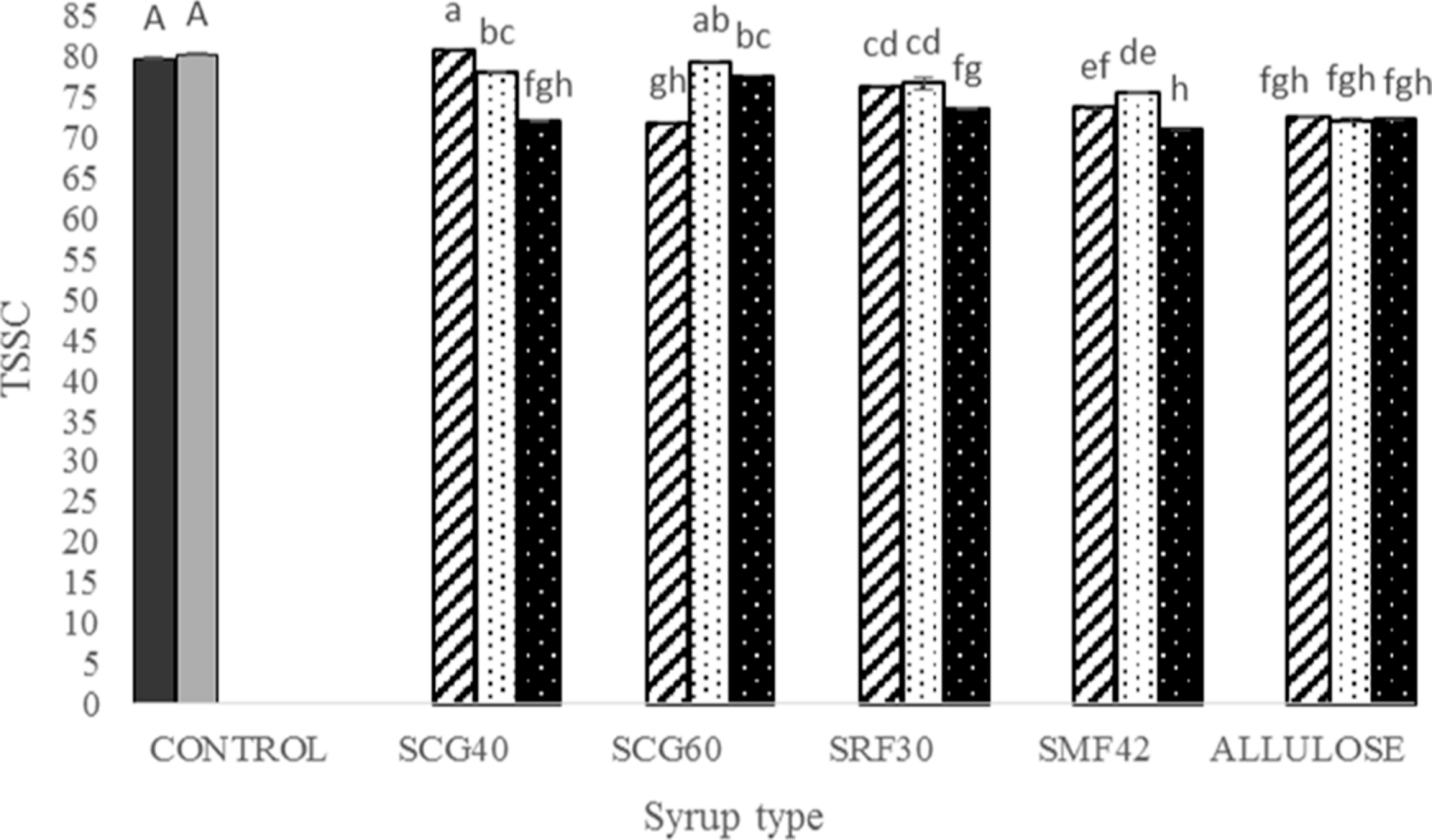
TSSC values (°) of control Turkish delights (COM: black shaded □ and SUC: gray shaded □) and Turkish delights containing different types of syrups at different concentrations (30%: black striped □, 45%: black dotted □, and 60%: white dotted ■). Different capital letters indicate significant difference (*p* < 0.05) for control samples (COM and SUC). Different small letters indicate significant difference (*p* < 0.05) for different types of syrup-containing samples with different syrup amounts (%). * Data were recorded with standard errors. Lowercase letters denote a significant difference between the samples at a 95% confidence level between the parameters. Analysis was done based on two replicates.

TSSC is defined as the weight (g) of total soluble solids in a 100 g solution, and it is expressed as (°Brix) unit.^36^ In most of the gummy candy formulations, TSSC values change in the range of 74–80°.^37^ A similar case is also valid for the Turkish delights which are classified as gummy candies. According to the Turkish legislation, the TSSC value of Turkish delights should be at least 80°.^2^ As shown in Figure 1, TSSC values of both control samples were found to be 80 and 80.2° for the commercial product purchased from the market (COM) and the product prepared under laboratory conditions (SUC), respectively. Both of these products were in accordance with the national legislation [Uslu et al. (2010)]. For the syrup and syrup/sucrose-containing samples, smaller TSSC values changing in the range of 71–79° were measured. Regardless of the syrup type, the lowest TSSC values were found for the samples containing only syrup [regardless of the syrup in their formulations (*p* < 0.05)]. Exceptionally, for the allulose syrup-containing samples, detectable changes were not observed when allulose syrup was substituted with 30, 45, and 60% sucrose, and for all these mentioned samples, similar TSSC values (∼72°) were obtained. TSSC values also might give an idea about the gel strength of the confectionery products. This case will be further discussed in oncoming sections.

### 3.2 MC Determination

**Figure 2:**
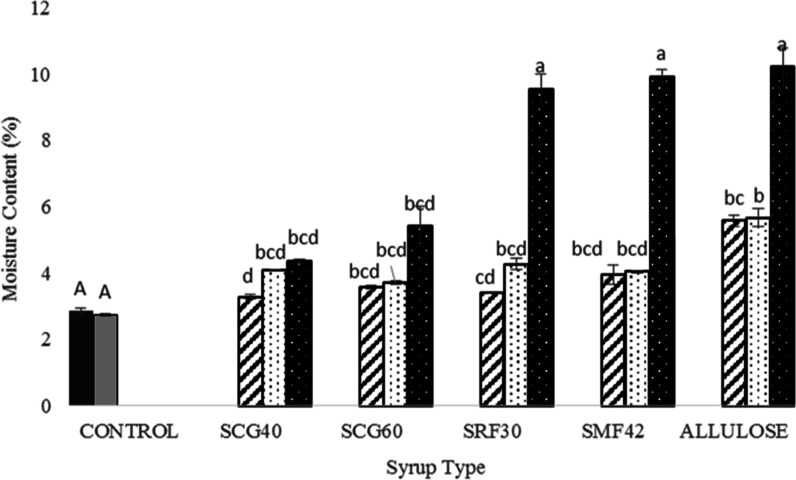
MC (%) of control Turkish delights (COM: black shaded □ and SUC: gray shaded □) and Turkish delights containing different types of syrups at different concentrations (30%: black stripped □, 45%: black dotted □, and 60%: white dotted ■). Different capital letters indicate a significant difference (*p* < 0.05) for control samples (COM and SUC). Different small letters indicate significant difference (*p* < 0.05) for different types of syrup-containing samples with different syrup amounts (%). * Data were recorded with standard errors. Lowercase letters denote a significant difference between the samples at a 95% confidence level between the parameters. Analysis was done based on two replicates.

Although water is not the main ingredient in most confectionery products, it has a vital role in terms of quality, shelf life, and manufacturing of the products.^4,37^ MC is also an important parameter for Turkish delights. As shown in Figure 2, similar and the lowest MC values were found for the control samples (COM and SUC). On the other hand, for the samples containing corn and allulose syrups, a detectable increase was found in the MC of the samples. Another important point that should be mentioned here was that as the syrup substitution increased from 30 to 60%, for the glucose syrup-containing samples (SCG40 and SCG60), detectable changes in MC were not observed (*p* > 0.05), while for the ones containing fructose/glucose syrup (SRF30 and SMF42) and allulose syrup in their formulation, a significant increase was observed when these types of syrups were used as the only sugar source (60%) compared to their counterparts including 30 and 45% syrups. Actually, this trend was an expected result since it was known that gummy candies produced by using a high amount of corn syrup easily pick up moisture due to their hygroscopic (water-binding) nature.^4^ Hygroscopic substances are also known as humectants which promote the retention of water.^4^ Ergun et al. (2010) stated that humectants are considered as molecules that contain hydroxyl groups having an affinity to form hydrogen bonds with water molecules. Hereby, they keep the confectionery products moist.^4^ In another study, it was also reported that these kinds of interactions are called “hydration” reactions and generally occur for all types of sugar.^9^ However, for the corn syrups with higher dextrose equivalent values, hydration occurs to a larger extent compared to the other types of corn syrups and sucrose.^4^ Coming back to our study, since SRF30-60 and SMF42-60 samples included only fructose/glucose-based corn syrup as the sugar source in their formulation, the highest MC (∼10%) of these samples was an expected result. On the other hand, for the Turkish delights containing only allulose syrup as the sugar source (allulose-60), the case is different. In previous studies, the effect of substitution of d-allulose was investigated for the starch-based^10^ and pectin-based^11^ soft candies, and it was found that as the d-allulose substitution increased in the formulations, the MC of the products decreased. This trend was attributed to the lower binding capacity of d-allulose,^38^ and in these studies, it was hypothesized that d-allulose-containing samples might have lost a substantial amount of water during cooking compared to their sucrose-containing counterparts.^10,11^ However, in this study, as the d-allulose syrup substitution increased, the MC of the samples increased significantly (*p* < 0.05). Moreover, despite having a low amount, allulose syrup utilized in the present study included 5% glucose. The presence of glucose probably enhanced the hygroscopicity of Turkish delights.

In the formulations containing both sucrose and syrups (30, 45%) detectable changes were not observed in MC (*p* > 0.05), probably due to the existence of sucrose having a less hygroscopic nature compared to corn syrups. This hypothesis was also supported by previous studies since it was indicated that the hydration of disaccharides increased in the order *sucrose < maltose < trehalose*.^38^ Since corn syrups are starch hydrolysis products which are rich in maltose and maltodextrin,^39^ they retain more water compared to sucrose, leading to higher MC in delights that included only corn syrup as the sugar source compared to their sucrose/syrup-containing counterparts.

It should also be highlighted that, according to the national legislation, the MC of Turkish delights should not exceed 16%.^40^ In this context, as shown in the data, all delights formulated in this study did not exceed this limit, even the ones with allulose syrups.

### 3.3 Color Analysis

The CIE color measurement model was used to calculate the L*, a*, and b* parameters of Turkish delights. L* indicates the lightness (which is adjustable between 0 and 100), and a* and b* factors are attributed to the red–green and yellow–blue axis, respectively.^41^

Color values of Turkish delights are given in Table 3. For the control samples (COM and SUC), significantly different results were obtained, and a detectable decrease was observed in all color values of the SUC sample compared to that in COM (*p* < 0.05). Although these two samples include the same sugar type (powder sucrose) in their formulation, these changes observed in color values might be attributed to the production methods. For example, SUC samples were formulated by using oil baths and their production methods mentioned in previous sections. However, since the COM sample is a commercial product that was purchased from the market, during its mass production, different processing methods that were applied might have affected their quality positively. For example, during the cooking of delights, some factories use pressurized vessels, while others use open vessels.^1^ It was reported that the inversion of sucrose in pressure cooking occurs faster than in open-vessel cooking.^1^ Therefore, pressurized vessels might have been used in the production of COM samples, leading to more successful inversion compared to SUC samples (produced at 125° in an oil bath), and higher amounts of sucrose might have been converted to glucose and fructose, leading to an increase in color values due to increased caramelization reactions.

**Table 3:** *L*, *a*, and *b* Values of Control Turkish Delights (COM and SUC) and Turkish Delights Containing Different Types of Syrups at Different Concentrations (30, 45, and 60%)^a^

sample	L*	a*	b*
CONTROL-1 (COM)	46.92 ± 0.03^A^	1.01^A^	2.31 ± 0.03^A^
CONTROL-2 (SUC)	30.98^B^	0.18^B^	0.98 ± 0.04^B^
SCG40-30	13.99^j^	0.15^gh^	0.03^f^
SCG40-45	29.80 ± 0.02^e^	0.06^i^	1.35^c^
SCG40-60	30.22 ± 0.01^e^	0.14^ghi^	0.39^ef^
SCG60-30	19.22 ± 0.01^j^	0.17^fgh^	0.07^f^
SCG60-45	35.34 ± 0.06^c^	0.20^fg^	0.96^cd^
SCG60-60	32.68 ± 0.70^d^	0.24^ef^	0.65 ± 0.35^de^
SRF30-30	20.86 ± 0.02^hd^	0.48^d^	2.36^ab^
SRF30-45	22.18 ± 0.02^h^	0.30^e^	0.23 ± 0.01^ef^
SRF30-60	41.26 ± 0.02^a^	0.53^d^	1.41^c^
SMF42-30	27.57^f^	0.22^efg^	0.47^ef^
SMF42-45	31.1^e^	0.11^hi^	0.59^de^
SMF42-60	25.47 ± 0.08^g^	0.19^fg^	0.28^ef^
ALLULOSE-30	39.26 ± 0.04^b^	0.87 ± 0.02^b^	2.11 ± 0.06^b^
ALLULOSE-45	38.56 ± 0.19^b^	1.12 ± 0.03^a^	2.79 ± 0.22^a^
ALLULOSE-60	24.54 ± 0.01^g^	0.71 ± 0.01^c^	1.24^c^

a Different capital letters indicate a significant difference (*p* < 0.05) for control samples (COM and SUC). Different small letters indicate a significant difference (*p* < 0.05) for different types of syrup-containing samples with different syrup amounts (%). * Data were recorded with standard errors. Lowercase letters denote a significant difference between the samples at a 95% confidence level between the parameters. Analysis was done based on two replicates.

As seen in Table 3, for the syrup-containing samples, the highest L* value was found for the SRF30-60 sample containing only SRF30 glucose/fructose syrup as the sugar source (*p* < 0.0.5). As indicated by Batu et al. (2016), lightness could be considered as an important quality parameter for Turkish delights.^40^ Therefore, the SRF30-60 sample has enhanced quality characteristics in terms of color values compared to its syrup-containing counterparts. However, it should be noted that its lightness value was still smaller than that of the original COM sample.

The highest a* and b* values were found for the allulose-45 sample which includes 45% allulose syrup and 15% powder sucrose (*p* < 0.05). Allulose-30 and allulose-60 also showed higher a* and b* values compared to the specimens including different types of glucose and glucose/fructose syrups. Since the a* and b* values show redness and yellowness, respectively, an increment in these values might be related to the enhancement of the caramelization reaction rate. Similar results were also obtained in previous studies. Ates et al. (2020) studied the effect of d-allulose substitution on pectin-based soft candies, and they found that increased color values are an indication of the occurrence of the caramelization reaction.^11^ In another study, the reactivity of allulose in caramelization reactions was investigated, and it was found that allulose is a highly reactive reducing sugar compared to glucose and fructose during caramelization reactions.^42^ Their findings were found to be in accordance with the results obtained in the present study.

### 3.4 Texture Profile Analysis

As seen in Table 4, control samples (COM and SUC) showed significantly different (*p* < 0.05) textural properties and hardness, cohesiveness, springiness, and gumminess results were all found to be smaller for the SUC samples compared to those for the COM samples. This case might be related to the higher retrogradation rate of the COM sample, leading to increased crystallinity and formation of harder samples compared to that of the SUC sample as will be discussed in the upcoming sections. Since the COM sample represents the commercial sample as mentioned previously and could not be analyzed just after production, an increased rate of retrogradation is not surprising for this sample compared to its SUC counterpart.

**Table 4:** Hardness, Adhesiveness, Cohesiveness, and Springiness Values of Control Turkish Delights (COM and SUC) and Turkish Delights Containing Different Types of Syrups at Different Concentrations (30, 45, and 60%)^a^

sample	hardness (N)	cohesiveness	springiness (mm)	gumminess (N)
CONTROL-1 (COM)	1.52 ± 0.02^A^	0.43 ± 0.02^A^	4.15 ± 0.25^A^	0.64 ± 0.03^A^
CONTROL-2 (SUC)	0.64^B^	0.19^B^	2.08 ± 0.04^B^	0.13 ± 0.04^B^
SCG40-30	1.47 ± 0.07^fg^	0.37^cdefg^	6.68 ± 0.45^abc^	0.54 ± 0.02^ghi^
SCG40-45	4.66 ± 0.01^a^	0.67^a^	7.01 ± 0.03^ab^	3.12 ± 0.04^a^
SCG40-60	2.60 ± 0.04^de^	0.34 ± 0.02^cdefg^	4.45 ± 0.01^cde^	0.53 ± 0.02^ef^
SCG60-30	1.70 ± 0.05^f^	0.31 ± 0.02^efg^	4.92 ± 0.08^cde^	0.52 ± 0.01^ghi^
SCG60-45	3.96 ± 0.12^b^	0.58 ± 0.01^ab^	6.72 ± 0.35^abc^	2.27 ± 0.03^b^
SCG60-60	1.92 ± 0.06^f^	0.34 ± 0.02^defg^	4.45 ± 0.01^def^	0.53 ± 0.02^ghi^
SRF30-30	2.75 ± 0.18^de^	0.32^efg^	4.55 ± 0.14^def^	0.89 ± 0.06^efg^
SRF30-45	3.35 ± 0.03^c^	0.44 ± 0.03^bcde^	6.25 ± 0.42^abcd^	1.47 ± 0.08^cd^
SRF30-60	0.79 ± 0.01^hi^	0.39 ± 0.02^cdef^	4.77 ± 0.19^def^	0.31 ± 0.01^hij^
SMF42-30	1.01^gh^	0.65 ± 0.04^a^	7.82^a^	0.66 ± 0.03^fgh^
SMF42-45	2.54 ± 0.09^e^	0.48 ± 0.02^bcd^	5.86 ± 0.26^bcd^	1.22 ± 0.09^de^
SMF42-60	3.09 ± 0.09^cd^	0.54 ± 0.02^ab^	6.29 ± 0.02^abcd^	1.65 ± 0.08^c^
ALLULOSE-30	0.31^ij^	0.25 ± 0.01^fg^	2.99 ± 0.17^f^	0.08^j^
ALLULOSE-45	0.19^j^	0.23 ± 0.01^g^	2.96 ± 0.32^f^	0.04^j^
ALLULOSE-60	0.57^hij^	0.49 ± 0.02^bc^	3.29 ± 0.09^ef^	0.23 ± 0.05^ij^

a Different capital letters indicate a significant difference (*p* < 0.05) for control samples (COM and SUC). Different small letters indicate a significant difference (*p* < 0.05) for different types of syrup-containing samples with different syrup amounts (%). * Data were recorded with standard errors. Lowercase letters denote a significant difference between the samples at a 95% confidence level between the parameters. Analysis was done based on two replicates.

Actually, although there are no certain rules that are determined by the Turkish legislation in terms of textural properties of the delights, it was explained that Turkish delights should be neither too hard nor too soft.^1^ However, as indicated by Batu and Kirmaci (2009), the elasticity of the delights could be considered as an important textural property.

Among the syrup-containing samples, the highest hardness, cohesiveness, springiness, and gumminess values were found for the SCG40-45 sample which contains 45% SCG40 glucose syrup and 15% sucrose in its formulation as a sugar source (*p* < 0.05). As an important outcome, for this sample, the highest springiness (7.01 mm) value and the highest cohesiveness (0.67) value were also obtained. Springiness is related to the elasticity of the sample, while cohesiveness is defined as the ability of the gel to hold its structure together, leading to the formation of a strong gel network that resists rupturing.^43^ Regarding these definitions, it could be deduced that these two parameters have the utmost importance to define the quality attributes of Turkish delights. It could be concluded that the SCG40-45 sample was found to be more elastic and shows a strong gel structure compared to its counterparts.

The lowest hardness, cohesiveness, springiness, and gumminess values were found for the allulose-containing samples, indicating weak gel formation for these samples. The hardness, cohesiveness, springiness, and gumminess values were found to be the lowest and similar for allulose-30 and allulose-45 (*p* < 0.05). Surprisingly, only the allulose-60 sample which solely included allulose syrup in its formulation was found to have higher hardness, cohesiveness, springiness, and gumminess values compared to its allulose syrup-/sucrose-containing counterparts. Most probably, for these three samples, this case was related to the lower water-binding ability of d-allulose compared to that of sucrose.^8−11,38^ As indicated in previous studies, due to its low hydration properties, d-allulose enhances gelatinization by providing more water for the starch molecules.^10^ Referring back to our data since the allulose-60 sample included only allulose syrup and there was no sucrose in its formulation, allulose might have bound water to a lesser extent, leading to provide more water for starch gelatinization and improved gel properties compared to the samples including sucrose and allulose syrup in their formulations (allulose-30 and allulose-45).

On the other hand, it was known that actually d-allulose and fructose exhibited similar water-binding properties, and both of them hydrated less compared to sucrose and other types of disaccharides.^38^ However, the hardness, cohesiveness, springiness, and gumminess values of fructose syrup-containing samples (SRF30 and SMF42) were found to be higher compared to those of allulose syrup-containing ones. This result was somehow contradictory. Rather than the water-binding ability of the sugars, for this case, another mechanism related to “caramelization reactions” seemed to be dominating. As indicated in previous sections, d-allulose is prone to caramelization reactions to a higher extent compared to the other types of sugar due to its reactivity.^42^ It was reported in most studies that, prior to the caramelization reaction, water forms as a result of the melting of the crystals.^44^ Therefore, at this point, it could be hypothesized that, due to this “water formation” that is related to the enhanced caramelization reaction rate, allulose syrup-containing samples might have softer and weaker gel network properties relative to the other delight samples.

In addition, an interesting trend was also observed in the textural properties of syrup-containing Turkish delights. The hardness values of glucose syrup-containing Turkish delights (SCG40 and SCG60) and SRF30 (fructose/glucose syrup-containing) samples increased significantly as the syrup substitution was increased from 30 to 45% (*p* < 0.05). However, when syrup substitution was increased from 45 to 60% (for totally syrup-containing samples), a sharp decrease in hardness was observed for the relevant samples. On the contrary, for the allulose syrup- and SMF42 (HFCS)-containing samples, hardness increased gradually by reaching the highest value for the totally syrup-containing samples in their formulation (60% substitution). This finding revealed that the use of the 45% syrup and 15% sucrose combination led to improved gel properties for the SCG40-, SCG60-, and SRF30-containing samples, while for the allulose- and SMF42-containing samples, the utilization of only 60% syrup in formulation (without sucrose) resulted in enhanced gel network properties. These different trends that were observed in the hardness values of the samples could be an indication of different interactions of syrup and sucrose that were used in the formulations.

To sum up, due to the distinct characteristics of sugar/syrup types, the utilization of different types of syrups in various amounts led to detectable changes in the textural properties of Turkish delights. It should be highlighted that, according to the syrup types that were used in the formulations, some properties may be desirable, while others may not.

### 3.5 *T*_2_ (Spin–Spin) Relaxation Spectra

In the present study, multiexponential analysis of decaying *T*_2_ curves was performed and two distinct proton populations (P1 and P2) with different relaxation times (*T*_2a_ and *T*_2b_) and different contributions (RAs) were detected for all samples, as seen in Table 5. Among these proton pools, P1 was generally attributed to the nonexchanging proton pool,^19^ and it was associated with rigid proton interactions that were not exposed to water,^8^ whereas P2 was thought to be associated with relatively more mobile water which was confined in the gel network.^19^ Therefore, RA_1_ (%) demonstrates the contribution of the nonexchanging proton pool, while RA_2_ (%) shows the contribution of signal coming from more mobile water that was entrapped in the gel network.

**Table 5:** *T*_2_ (Spin–Spin) Relaxation Spectrum Results of Control Turkish Delights (COM and SUC) and Turkish Delights Containing Different Types of Syrups at Different Concentrations (30, 45, and 60%)^a^

sample	*T*_2a_	*T*_2b_	RA_1_ (%)	RA_2_ (%)
CONTROL-1 (COM)	0.12 ± 0.01^B^	1.05 ± 0.07^A^	80 ± 1.41^A^	20 ± 1.41^B^
CONTROL-2 (SUC)	0.23^A^	0.83^A^	40^B^	60^A^
SCG40-30	0.17^d^	1.05 ± 0.05^ef^	40 ± 1.77^cde^	60 ± 0.02^cde^
SCG40-45	0.09^d^	0.67 ± 0.02^ef^	47 ± 1.06^bcd^	53 ± 1.06^def^
SCG40-60	0.07^d^	0.59^f^	59^a^	41^g^
SCG60-30	0.26 ± 0.05^d^	1.33 ± 0.05^de^	29 ± 0.35^fg^	71 ± 0.35^ab^
SCG60-45	0.14^d^	0.78 ± 0.02^ef^	47 ± 1.77^bcd^	53 ± 1.77^def^
SCG60-60	0.09^d^	0.55 ± 0.01^f^	54 ± 0.35^ab^	46 ± 0.35^fg^
SRF30-30	0.26^d^	1.28 ± 0.02^de^	35 ± 1.41^efg^	65 ± 1.41^abc^
SRF30-45	0.18^d^	0.83 ± 0.03^ef^	40 ± 1.77^cde^	60 ± 1.77^cde^
SRF30-60	0.78 ± 0.01^c^	2.87 ± 0.05^ab^	29^fg^	71^ab^
SMF42-30	0.83 ± 0.07^c^	1.80 ± 0.1^cd^	48 ± 3.18^bc^	52 ± 3.18^ef^
SMF42-45	1.09 ± 0.02^ab^	2.26 ± 0.06^bc^	49 ± 0.35^bc^	51 ± 0.35^ef^
SMF42-60	1.20 ± 0.08^a^	2.77 ± 0.27^ab^	41 ± 0.71^cde^	59 ± 0.71^cde^
ALLULOSE-30	0.79 ± 0.01^c^	2.36 ± 0.05^bc^	26 ± 0.71^g^	74 ± 0.71^a^
ALLULOSE-45	0.89^bc^	2.56 ± 0.05^b^	37 ± 0.71^def^	63 ± 0.71^bcd^
ALLULOSE-60	0.85^c^	3.34 ± 0.04^a^	33 ± 0.35^efg^	67 ± 0.35^abc^

a Different capital letters indicate a significant difference (*p* < 0.05) for control samples (COM and SUC). Different small letters indicate a significant difference (*p* < 0.05) for different types of syrup-containing samples with different syrup amounts (%). * Data were recorded with standard errors. Lowercase letters denote a significant difference between the samples at a 95% confidence level between the parameters. Analysis was done based on two replicates.

Ilhan et al. (2020) characterized starch-based gummy candy productions by utilizing *T*_2_ NMR relaxometry, and they found that compartments with the lowest relaxation times were generally related to solid–solid interactions which might stem from sugar–starch or sugar–sugar interactions.^10^ Since Turkish delights formulated in this study are also starch-based confectionery products, a similar case is also valid for our study. As seen in Table 5, among the control samples, the lowest *T*_2a_ (*T*_2_ relaxation times of the P1) relaxation time was found for the original COM delights (*p* < 0.05), while relatively higher *T*_2a_ relaxation times were found for the SUC samples. Contrary to *T*_2a_ values, the highest RA_1_ was also found for the COM sample, indicating enhanced solid–solid interactions and the formation of a strong gel network for this product. This case might be related to the enhanced starch retrogradation that was observed for the COM sample compared to that for the SUC sample. As a result of the higher retrogradation rate, a higher crystallinity degree was also observed for the COM sample compared to that for the SUC sample (will further be discussed in the “X-ray Diffraction Analysis” section). Since crystal structures hold less water, shorter *T*_2a_ relaxation times and a higher peak area of P1 (RA_1_) were detected for the COM sample compared to that for the SUC sample. Considering the same analogy, a significant decrease in RA_2_ of the COM sample compared to that in the RA_2_ of the SUC sample is also not surprising since the second compartment (P2) was attributed to the water having higher mobility than was entrapped in the gel network. As indicated previously in the “Texture Profile Analysis” section, the hardness values of the SUC sample were found to be significantly smaller than those of the COM sample, indicating the existence of a high amount of water in the confined gel network, which might have not been removed during cooking because of inadequate process conditions, leading to weak gel formation. Another finding about these control samples is that very similar *T*_2b_ relaxation times were found for the COM and SUC delights, and detectable changes were not observed (*p* > 0.05). This situation is expected because it was thought that the sugar type directly affects the relaxation times of P2 (*T*_2b_) due to the existence of dissolved sugars in water that was confined in the gel network.^8^ Since both of these original Turkish delights (COM and SUC) contain only sucrose as the sugar source, similar *T*_2b_ relaxation times were obtained.

When corn syrups were used in the Turkish delight formulations, detectable changes in both relaxation times and RAs of peaks were observed compared to control samples. For the samples formulated with glucose syrup (SCG40 and SCG60), increased syrup substitution did not lead to detectable changes in *T*_2a_ relaxation times, indicating that glucose syrup substitution did change the solid–solid interactions significantly (*p* > 0.05). RA_1_ for these samples increased significantly when the syrup substitution reached 60%. As the syrup substitution was increased for these samples, a decrease in *T*_2b_ and RA_2_ was observed as expected because corn syrups were known for their humectant (*more water binding*) properties due to the substantial amount of maltose as mentioned previously. Therefore, for SCG40 and SCG60 samples, an increased amount of syrup led to a decrease in the mobility of water, resulting in a decrease in *T*_2b_ and RA_2_ values.

Among the fructose/glucose syrup-containing samples (SRF30 and SMF42), as the syrup substitution was increased, *T*_2a_ relaxation times also increased by reaching the maximum value for the samples containing only syrup (*p* < 0.05). On the other hand, RA_1_ decreased gradually for these samples as the substitution of syrup increased. This case was most probably related to the decrease in solid–solid interactions. Actually, this situation was found to be in accordance with the TSSC values. Referring back to TSSC values as illustrated in Figure 1, the TSSC decreased significantly for the SRF30- and SMF42-containing samples at a 60% substitution level. Therefore, it was hypothesized that the decrease in TSSC led to a decrease in solid–solid interactions, resulting in an increase in *T*_2a_ and an increase in RA_2_. Considering the whole data set, it was also found that the TSSC and *T*_2a_ relaxation times were found to be negatively correlated (*r* = −0.64, *p* < 0.05), proving the hypothesis about TSSC and *T*_2a_ relaxation time constants mentioned previously. Another important observation about the SRF30- and SMF42-containing samples was the ascending trend of *T*_2b_ and RA_2_ as the syrup substitution was increased. This case might be related to the enhanced caramelization reaction rate occurring for these samples. According to previous studies, it is known that the contribution of fructose to browning development is generally higher than that of glucose during the caramelization reactions.^45^ Therefore, it is probable that fructose syrup-containing samples might have caramelized more than glucose syrup-containing ones. In addition, as mentioned previously, prior to the caramelization reaction, a new water fraction forms as a result of the melting of the crystals.^44^ Due to this newly formed water pool, the water mobility of P2 might have increased, giving rise to an increase in *T*_2b_ and RA_2_.

For the allulose syrup-containing samples, a steady trend was observed in *T*_2a_ relaxation times. Since the TSSC of these samples did not change as the syrup substitution was increased, this result in *T*_2a_ relaxation times was not surprising. On the other hand, increased allulose syrup substitution led to an increase in *T*_2b_. This result might be again attributed to the enhanced caramelization rate of allulose syrup as in the case of fructose syrups that was mentioned above. It is also worth mentioning that the highest RA_2_ and *T*_2b_ results were found for these samples among all syrup-containing samples, indicating that the mobility of water in the gel network is also the highest for allulose syrup-containing samples. This outcome might stem from the less interaction of d-allulose with water compared to other types of sugars such as glucose, fructose, and sucrose, as indicated in a previous study.^46^

### 3.6 XRD Analysis

**Figure 3:**
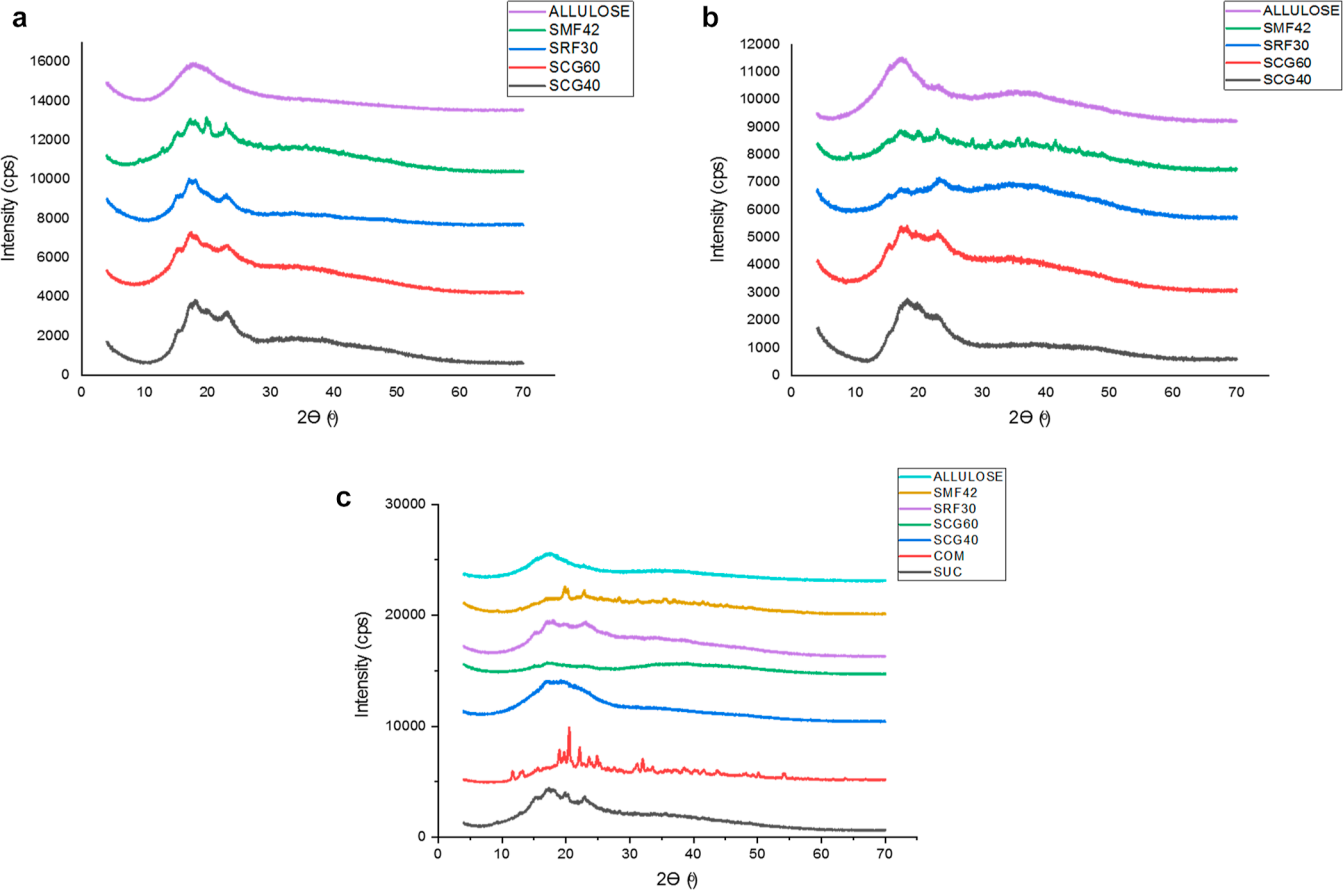
(a) XRD patterns of control (COM and SUC) and different types of syrup (30% concentration)-containing Turkish delights. (b) XRD patterns of Turkish delights containing different types of syrups (45% syrup concentration). (c) XRD patterns of Turkish delights containing different types of syrups (60% syrup concentration).

**Figure 4:**
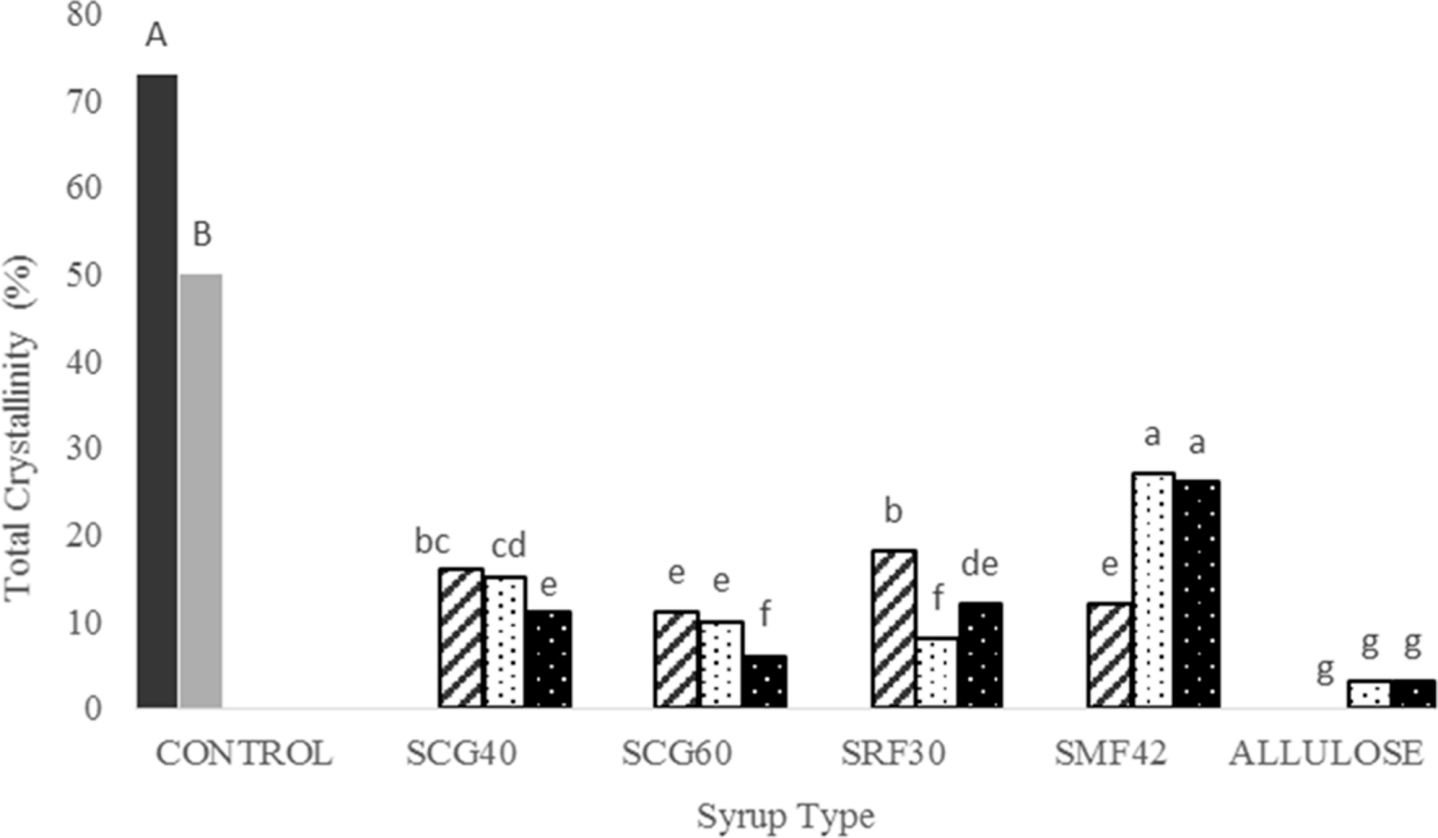
Total crystallinity (%) of control Turkish delights (COM: black shaded □ and SUC: gray shaded □) and Turkish delights containing a different type of syrups at different concentrations (30%: black stripped □, 45%: black dotted □, and 60%: white dotted ■).

**Figure 5:**
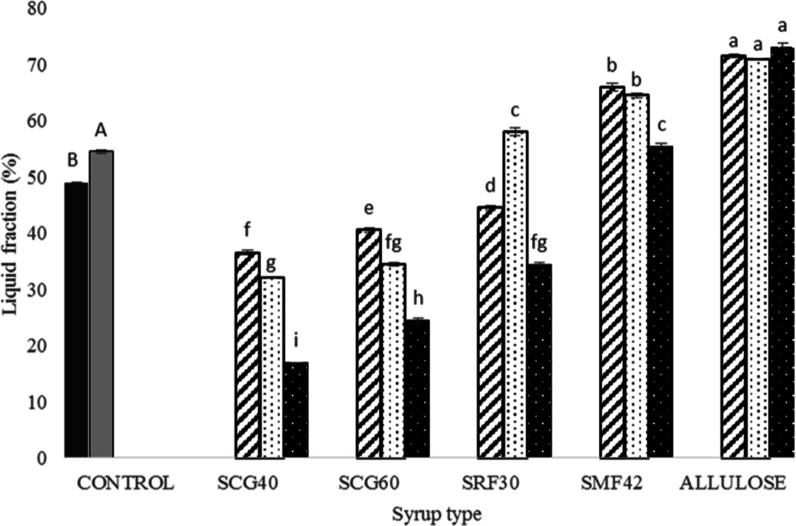
Liquid fraction (%) results of control Turkish delights (COM: black shaded □ and SUC: gray shaded □) and Turkish delights containing different types of syrups at different concentrations (30%: black stripped □, 45%: black dotted □, and 60%: white dotted ■). Different capital letters indicate a significant difference (*p* < 0.05) for control samples (COM and SUC). Different small letters indicate a significant difference (*p* < 0.05) for different types of syrup-containing samples with different syrup amounts (%). * Data were recorded with standard errors. Lowercase letters denote a significant difference between the samples at a 95% confidence level between the parameters. Analysis was done based on two replicates.

XRD analysis of Turkish delights was performed, and patterns of the samples obtained are shown in Figure 3a–c. While interpreting the XRD pattern, it is important to note that the narrower and more concentrated peaks are associated with the crystal regions, whereas the larger and less dense peaks are related to the amorphous regions.^8,10^ In order to determine the crystalline peaks more clearly in the X-ray pattern, Turkish delights were dried at room temperature for 6 months, as mentioned in the previous sections. In this context, as seen in Figure 3c, it could be clearly stated that COM samples are the ones with the highest crystallinity degree compared to SUC and other totally syrup-containing counterparts (60% syrup substitution) by demonstrating various sharper and narrower peaks in their X-ray pattern. This case is an expected result because corn syrups have a crystallization inhibition nature as mentioned previously, and that is why manufacturers prefer to use corn syrups in the production of Turkish delights. The interesting outcome here that should be mentioned is that, among the control samples, the crystallinity of COM samples was found to be higher (73%) compared to that of SUC (50%) even though they both contain only powder sucrose as the sugar source regarding the total crystallinity (%) degree calculation, as presented in Figure 4. This case might have stemmed from the different retrogradation rates between the COM and SUC samples, as mentioned previously. Most probably, the COM samples hold less water due to the exposure of higher retrogradation, as also validated by NMR relaxation spectra since detectable changes in water pools were observed for these COM and SUC samples. Not only starch retrogradation but also sucrose crystallization in COM samples might have led to various sharp and narrow peaks in the X-ray spectra (Figure 3c) and the highest crystallinity degree (73%) among all-Turkish delight samples. The final MC of the products that were stored for 6 months was not measured, but the liquid fraction (%) of these samples was also determined by also using low-field TD-NMR relaxometry. As seen in Figure 5, the final liquid fraction (%) for the COM sample was also found to be significantly lower compared to that for the SUC sample which might be the result of the higher crystal amount and consequently the less liquid amount found in the COM sample compared to that in the SUC sample. Liquid fraction measurement by TD-NMR will be also discussed in detail in the later section.

Coming back to the crystallization results of the delight samples, for all corn syrup-containing samples, a dramatic decrease was observed in crystallinity degrees at all substitution levels (30, 45, and 60%) compared to the control ones, and their crystallinity degree was found to be in the range of 8–27% (Figure 4). Herein, only the crystallinity degree of 30% allulose syrup containing was not calculated since it showed a totally amorphous pattern (Figure 3a). 45 and 60% allulose syrup-containing samples’ crystallinity were also found to be very small and similar (∼3%). Detailed information about the allulose syrup-containing samples will be discussed later. In addition, as the syrup concentration increased from 30 to 60%, enlargement of the bottom width of individual peaks was observed, which could be an indication of the decreased crystal size of samples containing a high amount of syrup in their formulation. This result was in accordance with previous studies since a similar “enlargement of the bottom width of peaks” was observed in the XRD patterns of powder glucose and lactose when they were freeze-dried, demonstrating a less crystalline nature due to the decreased crystalline size.^32^

As indicated above, the crystallinity of the corn syrup-containing samples (SCG40, SCG60, SRF30, and SMF42) at all substitution levels (30, 45, and 60%) was found to be significantly different and less compared to the control samples (COM and SUC), which was also validated by their XRD patterns (Figure 3a–c). This is an expected result actually since the growth of sucrose and nucleation could be eliminated by using corn syrup.^47^ Therefore, manufacturers generally use corn syrups as a crystallization inhibitor in order to control the level of crystallization.^3^

Among the glucose syrup (SCG40 and SCG60)-containing samples, relatively lower crystallinity values were observed, and for these delights, no detectable changes were observed in the crystallinity values when the syrup substitution was increased from 30 to 45%, while at a 60% substitution level, crystallinity reached its lowest value for both syrups. Among the only corn syrup-including samples (60%), SCG40 and SCG60 were found to have the lowest crystallinity degree compared to the other corn syrup-containing samples.

For the glucose/fructose syrup (SRF30 and SMF42)-containing samples, relatively higher crystallinity degrees were obtained compared to other samples including glucose syrup in their formulation (*p* < 0.05). Especially, for the HFCS-containing samples (SMF42), the crystallinity degree increased as the syrup substitution was increased and reached its maximum value at 45 and 60% substitution (*p* < 0.05). Herein, a contradiction exists since the crystallization degree is expected to decrease as the amount of corn syrup increases in the formulation. This outcome might be attributed to the existence of a high amount of glucose and fructose in the HFCS (SMF42). Having lower melting points than sucrose, glucose and fructose are considered as potential solvents for crystalline sucrose.^44^ Therefore, in the present study, glucose and fructose found in 30% SMF42-containing samples might have acted as a solvent for the sucrose crystals, leading to the formation of Turkish delights with a less crystalline nature. Another important point about the glucose/fructose syrup-containing samples is that, when the syrup amount is dominant in the formulations (at 60% substitution), the SMF42 samples had a higher crystallinity degree than SRF30 samples, although both of them are composed of syrups containing glucose and fructose. Herein, the important effect that led to this situation might be the different ratios of glucose/fructose found in SRF30 and SMF42 syrups. In previous studies, the crystallization rate of different honey samples was studied and they classified crystallization rates of honey samples according to the different fructose/glucose (F/G) ratios they contained.^48^ In their study, it was indicated that when F/G < 1.11, fast crystallization occurs, while a F/G > 1.33 results in a slower crystallization rate in honey samples.^48^ Since corn syrups (SRF30 and SMF42) utilized in the present study had very similar content to honey samples in terms of the glucose/fructose amount they contain, a very similar analogy could be also used in our study. According to this hypothesis, since the SRF30 syrup had a higher F/G ratio (1.39) compared to the SMF42 syrup (F/G: 0.82), slower crystallization might have occurred for SRF30 relative to that for SMF42, leading to the formation of fewer crystal amounts for the Turkish delights containing totally SRF30 syrup as the sugar source compared to the ones including solely the SMF42 syrup as the sugar source in its formulation, as also confirmed by XRD patterns (Figure 3c) and total crystallinity degree (%) calculations (Figure 4).

The crystallization inhibition behavior of the allulose syrup was found to be more pronounced compared to corn syrups for Turkish delights. As seen clearly in XRD patterns (Figure 3a–c) and total crystallinity degree (%) (Figure 4), allulose syrup-containing samples were found to have the least crystallinity among the all-Turkish delight samples (*p* < 0.05). Surprisingly, the allulose-30 sample which contains 30% sucrose and 30% allulose syrup demonstrated a totally “amorphous halo pattern” shape in its XRD pattern (Figure 3a), and no sharp crystalline peaks could be detected. Herein, the hypothesis that was proposed for the fructose syrups might be also valid since d-allulose is a C-3 epimer or fructose and shows very similar characteristics to fructose.^8^ For the other allulose syrup-containing samples (30 and 60%), the crystallinity (%) values were also found to be very low (3%), proving the power of d-allulose for inhibiting the crystallinity tendency of Turkish delights in accordance with the previous studies.^8,10^ For the allulose-45 and allulose-60 samples, in addition to the peak that appeared at 17°, which is associated with starch retrogradation^49^ and observed in all-Turkish delight samples in our study, only a small crystalline peak appeared at 23° which could be attributed to allulose crystals, as indicated by Ilhan et al. (2020). Therefore, it could be concluded that the d-allulose syrup retarded not only sucrose crystal formation but also starch retrogradation in Turkish delights.

At the first glance, such a high crystallization tendency of the allulose syrup may seem advantageous regarding the quality of Turkish delights. However, such a low crystallinity and even an amorphous structure is not a desirable characteristic for Turkish delights as indicated in previous studies.^1^ Although there is no detailed study examining the crystallinity properties of Turkish delights, a similar case is also valid for some other types of confectionery products such as fondants and fudges.^3^ As indicated by Porter and Hartel (2013), in order to provide a proper mouth feel and the structural shape of fondants and fudges, a certain amount of sucrose crystallization is required.^3^ Coming back to our study, it is worth noting that allulose syrup-containing samples had a very soft structure, as mentioned previously in the “Texture Profile Analysis” part. Even at the end of 6 months of storage, they remained soft, but because of this increased softness, they could not preserve even their shape, most probably due to their very low crystallinity value. As indicated in previous studies, although allulose is considered as a promising sugar replacement due to its low calorie value, it has a very strong plasticizing capacity leading to a drastic decrease in glass transition temperature (*T*_g_) and the formation of sticky products.^39^ This case might have occurred for our case in the production of Turkish delights, and due to the strong plasticizing effect of the allulose syrup, sucrose crystals might have been prone to melting and a totally amorphous structure was obtained even for the samples including an equal amount of allulose syrup (30%) and powder sucrose (30%), leading to the formation of very soft and sticky products, which is an undesirable case for Turkish delights.

It is worth mentioning that for some types of confectionery products, too little crystalline sucrose may result in a too soft structure, leading to a loss of its shape, while too much sucrose leads to the formation of dry and hard candy.^3^ This case is also valid for Turkish delights, and both the high crystallinity of original COM and SUC samples (73 and 50%, respectively) and the lowest value of crystallinity (3%) (allulose-45 and allulose-60), and even a totally amorphous structure (allulose-30), are not desirable characteristics of Turkish delights. At this point, HFCS (SMF42) could be considered as more advantageous as it gives a desirable crystalline degree (changing in the range of 12–27%) to the Turkish delights.

To sum up, XRD analysis seems like a perfect tool to detect the crystallinity of Turkish delights containing different types and amounts of syrups, although it has some disadvantages such as human judgment in peak analysis^50^ and its time-consuming nature.

### 3.7 Liquid Fraction (%) Measurements through MSE

As indicated previously, as well as by crystal content determination, the estimation of the liquid fraction in food systems is important since basic thermodynamic changes occurring during the crystallization process are strongly related to the changes in the concentration of the liquid phase.^51^ At this point, the importance of low-field TD-NMR is revealed due to its power to determine the solid–liquid ratio in foods and solid fat content determination.^52^ In various studies, the basic FID pulse sequence was generally utilized to define solid–liquid fractions and the crystallization behavior of food systems.^3,50,51,53^ Unlike the classical FID approach, liquid fractions (%) of Turkish delight samples were found by using the MSE sequence as explained in detail in the study of Grunin et al. (2019). Similar to the liquid fractions, second moment values (*M*_2_) which are related to the crystallinity of the samples were also found by using the same pulse sequence again by referring to the same study,^32^ as indicated in the oncoming sections.

First, it is worth mentioning that, in the present study, the liquid fraction (%) and second moment (*M*_2_) values obtained from the TD-NMR experiments are not the exact liquid and crystalline content values, but they provide a quick and easy method to estimate the crystal and liquid content in the samples by using Relax8 software.^32^ Moreover, as mentioned previously, these methods were applied to the dried form of the samples.

Liquid fractions (%) of Turkish delights samples are demonstrated in Figure 5, and as clearly seen, the use of different types and concentrations of syrup led to detectable changes (*p* < 0.05) excluding the allulose syrup-containing samples because, for this group, increasing syrup concentration did not result in any changes in the liquid fraction. As mentioned previously, although the original Turkish delights (COM and SUC) include the same type of sugar (powder sucrose) in their formulation, the liquid fractions they contained were found to be significantly different (*p* < 0.05), most likely due to the different production methods they were exposed to. Since the crystallinity of the COM sample is significantly higher than that of the SUC one and knowing the fact that “crystal structures hold less water”,^8^ it could be considered as an expected outcome.

Among the syrup-containing samples, the lowest liquid fraction (%) was detected for the glucose syrup-containing ones (SCG40 and SCG60), whereas the highest liquid fraction was obtained for the allulose syrup-containing samples (*p* < 0.05). This case might be explained by the decreased mobility of the glucose syrup-containing samples, while for the allulose and glucose/fructose syrup-containing samples, the mobility of water in the confined region might be increased, leading to a higher liquid fraction for these samples. It is a very well-known fact that the powder form of allulose retards the retrogradation by increasing starch–water interactions and leads to the entrapment of more water in the gel network.^10^ Therefore, a very similar approach is also valid for our study, so it was hypothesized that the allulose syrup also inhibits the retrogradation and sucrose crystallization, leading to increased water mobility in the gel network and resulting in the highest liquid fraction for these samples. A similar case is also valid for the SRF30 and SMF42 syrup-containing samples, and the high amount of fructose found in syrups might have also retarded retrogradation since allulose and fructose have very similar properties, as mentioned in previous sections.

In addition to the retrogradation inhibition properties, the higher reactivity of allulose and fructose in the caramelization reaction might have resulted in such a high liquid fraction for allulose, SRF30, and SMF42 syrup-containing samples. As mentioned previously by Roos et al. (2013), prior to the caramelization reaction, an additional water fraction is formed as a result of the melting of the crystals.^44^ This might have led to the formation of new proton pools and led to a detectable increase in the allulose fructose syrup-containing Turkish delights’ liquid fraction, as measured by TD-NMR.

### 3.8 Second Moment (*M*_2_) Measurements through MSE

The second moment (*M*_2_) indicates the strength of dipolar interactions where the rigid protons are involved, and for this reason, it inversely correlates with the molecular mobility of the rigid fractions.^34^ Therefore, it could be deduced that *M*_2_ is directly related to the mobility of the proton fractions. Knowing that crystallization is a phase transition accompanied by a change in molecular mobility,^54^*M*_2_ is also widely used to estimate the crystallinity content of the samples. It was observed that a higher crystallinity resulted in a step increase in *M*_2_ of dairy powders^54,55^ and powder sugars,^32^ enabling researchers to discriminate the “more mobile” amorphous molecules from the crystalline ones. Crystallization determination was also performed for the confectionery products by using basic FID,^3,50,51^ and in these studies, due to the “dead time” problem, the correction factor was used to estimate the crystalline content. The importance of the MSE sequence reveals that at this point since there is no need for a correction factor, it allows the detection of the signal coming from the solid fraction.^32^ Although MSE is widely used to determine the crystal/amorphous fraction in polymers^28,30^ and structural changes in cellulose during water absorption,^31^ very few studies exist in the literature examining the applications of MSE in food systems. Until now, it was only utilized to monitor honey crystallization and melting^33^ as a food system. To the best of our knowledge, there is no study in the literature examining the crystallinity of confectionery products by using the MSE pulse sequence as a nonconventional and novel TD-NMR technique. Therefore, in the present study, the crystallization of Turkish delights was first studied with the help of the MSE sequence and its power as an alternative to common crystallinity determination methods such as XRD was examined as a quality detection tool.

**Figure 6:**
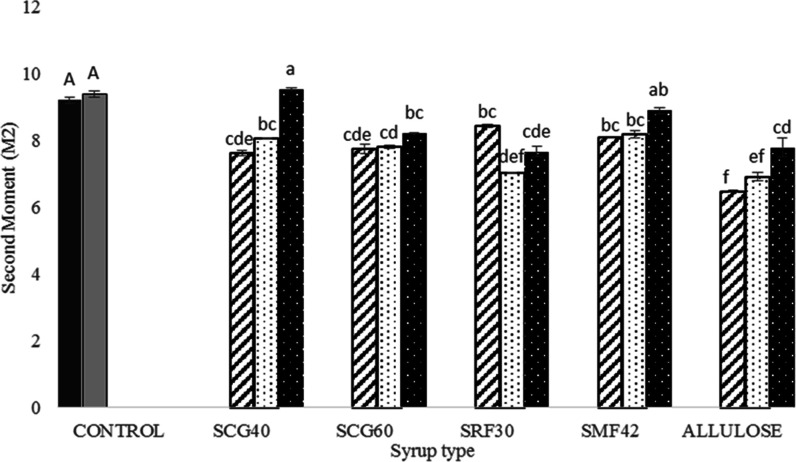
Second moment (*M*_2_) results of control Turkish delights (COM: black shaded □ and SUC: gray shaded □) and Turkish delights containing different types of syrups at different concentrations (30%: black stripped □, 45%: black dotted □, and 60%: white dotted ■). Different capital letters indicate a significant difference (*p* < 0.05) for control samples (COM and SUC). Different small letters indicate a significant difference (*p* < 0.05) for different types of syrup-containing samples with different syrup amounts (%). * Data were recorded with standard errors. Lowercase letters denote a significant difference between the samples at a 95% confidence level between the parameters. Analysis was done based on two replicates.

As shown in Figure 6, for the control samples (COM and SUC), very similar second moment (*M*_2_) values (9.21 × 10^9^ and 9.39 × 10^9^ s^–2^, respectively) were observed compared to the corn syrup-containing ones. These samples include only powder sucrose, and due to the higher dipolar contribution of sucrose,^8^ higher *M*_2_ values were obtained for these samples.

For the syrup-containing samples, relatively smaller *M*_2_ values were obtained as expected since low *M*_2_ is directly proportional to the crystallinity, as mentioned previously. Herein, the only exceptional sample is SCG40-60, which contains solely SCG-40 glucose syrup (60%) as the sugar source. Surprisingly, for this sample, among the all-Turkish delights, the highest *M*_2_ (∼9.53 × 10^9^ s^–2^) value was observed (*p* < 0.05). Although it has less crystallinity (11%) than the original samples, such a high second moment (*M*_2_) of this sample could be explained by the viscosity effect rather than crystallinity. Knowing that the SCG-40 syrup has the highest viscosity due to the inclusion of a high amount of starch hydrolysis products such as maltodextrin, its mobility might have been diminished more compared to other syrup types. Since *M*_2_ is inversely correlated with the mobility of rigid proton fractions,^34^ due to the decreased mobility of rigid protons in SCG40-60 samples, the highest *M*_2_ value might have been obtained for this sample. Therefore, it is worth mentioning that the *M*_2_ values obtained through MSE cannot be always related to the crystallinity degree but can be also associated with other types of effects such as viscosity.

The smallest *M*_2_ value (∼6.47 × 10^9^ s^–2^) was obtained for the sample including 30% allulose syrup in its formulation. Remembering that this sample was the one which had a totally amorphous structure, such a low *M*_2_ value was not surprising.

When all second moment (*M*_2_) results were considered, the utilization of different types of syrups at different concentration levels led to detectable changes in *M*_2_ results. Moreover, it was found that there was a significant correlation between *M*_2_ values and the total crystallinity degree (%) obtained through XRD analysis (*r* = 0.67, *p* < 0.05). Therefore, it could be concluded that the second moment values measured through TD-NMR by using the MSE pulse sequence might be a possible and easier alternative to XRD analysis to detect the crystallinity of Turkish delights including different types and amounts of sugar sources.

This study was built on three main purposes. The first one was to examine the effect of different types of corn syrups (glucose and glucose/fructose syrups) and allulose syrup substitution in Turkish delights by using important quality parameters such as TSSC, MC, color, and textural parameters (hardness, springiness, adhesiveness, etc.). The second one was to explain the distinct solid–solid and polymer–water interaction in various types of Turkish delights including different types and amounts in their formulations by using TD-NMR through the *T*_2_ relaxation spectra. The third and last objective of this study was to use the liquid fraction (%) and second moment (*M*_2_) by using TD-NMR through the MSE sequence as a nonconventional NMR technique to predict the crystallinity of Turkish delights as an easy and time-saving method compared to the commonly used XRD method.

Especially, some physical properties such as hardness, TSSC, and color values were found to be directly related to water mobility in the samples. This relation was observed as changes in *T*_2a_ and *T*_2b_ relaxation times and RA_1_ and RA_2_ of the *T*_2_ relaxation spectra, indicating that the relaxation spectra might give an idea about the physical properties of Turkish delight samples. In addition, crystallinity values (%) obtained through XRD analysis and *M*_2_ values obtained through TD-NMR experiments were found to be highly correlated (*r* = 0.67, *p* < 0.05), showing that TD-NMR can be a great alternative to XRD techniques.

The results clearly indicated that a very soft texture and weak gel formation were obtained for the allulose syrup-containing samples, resulting in poor textural properties, as validated by the *T*_2_ relaxation spectrum results. On the other hand, certain types of samples containing corn syrups were found to have superior properties compared to the original samples in terms of color and texture. In addition, for the crystallinity measurements, well-correlated results clearly indicated that nonconventional methods by using the MSE sequence might be a promising alternative due to the time-saving and less-laborious nature of the low-resolution TD-NMR technique compared to the commonly used time-consuming XRD technique which requires careful human judgment in peak analysis. The authors believe that the present study will pave the way for the utilization of both conventional and nonconventional methods of TD-NMR in the confectionery industry and R&D laboratories as an alternative quality detection tool.

## Supporting Information Available

Representative TPA curve for the SCG40-60 sample (PDF)
