# Supplementary figures and images for: Effect of Different Syrup Types on Turkish Delights (Lokum): A TD-NMR Relaxometry Study
Source: ACS Food Sci Technol. 2022 Nov 30;2(12):1819–31. doi: 10.1021/acsfoodscitech.2c00222 (PMC9775206; doi:10.1021/acsfoodscitech.2c00222)

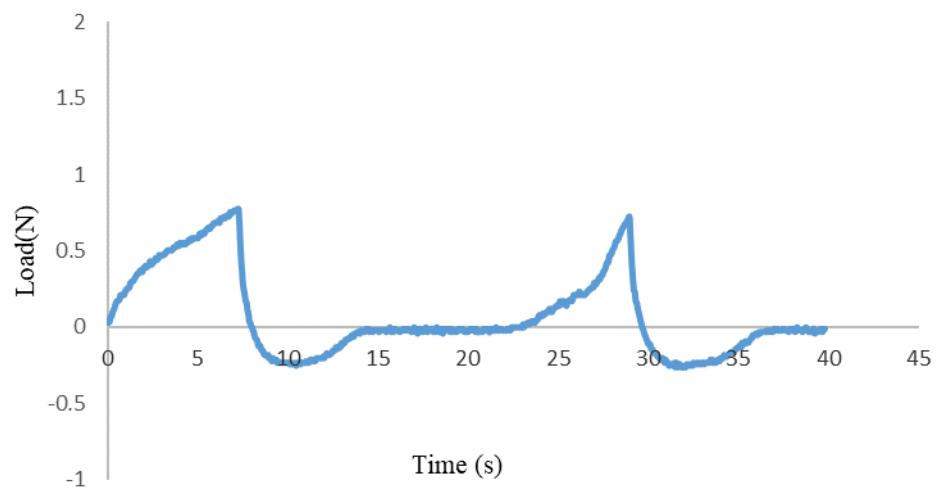

**Fig. S:** Representative TPA curve for the SCG40-60 sample

Supplement: Supplementary file 1 — fs2c00222_si_001.pdf [file fs2c00222_si_001.pdf]
